# FGFR3_△7–9_ promotes tumor progression via the phosphorylation and destabilization of ten-eleven translocation-2 in human hepatocellular carcinoma

**DOI:** 10.1038/s41419-020-03089-2

**Published:** 2020-10-23

**Authors:** Zhijian Jin, Haoran Feng, Juyong Liang, Xiaoqian Jing, Qiwu Zhao, Ling Zhan, Baiyong Shen, Xi Cheng, Liping Su, Weihua Qiu

**Affiliations:** 1 Department of General Surgery, Ruijin Hospital, Shanghai Jiao Tong University School of Medicine, Shanghai, 200025 China; 2 Department of Surgery, Shanghai Key Laboratory of Gastric Neoplasms, Shanghai Institute of Digestive Surgery, Ruijin Hospital, Shanghai Jiao Tong University School of Medicine, Shanghai, China; 3 Department of General Surgery, Ruijin North Hospital, Shanghai Jiao Tong University School of Medicine, Shanghai, China; 4 Shanghai Institute of Digestive Surgery, Ruijin Hospital, Shanghai Jiao Tong University School of Medicine, Shanghai, 200025 China

**Keywords:** Oncogenes, Growth factor signalling

## Abstract

Overexpression of fibroblast growth factor receptor 3 (FGFR3) correlates with more severe clinical features of hepatocellular carcinoma (HCC). Our previous study has shown that FGFR3_∆7–9_, a novel splicing mutation of FGFR3, contributes significantly to HCC malignant character, but the epigenetic mechanism is still elusive. In this study, through mass spectrometry and co-immunoprecipitation studies, we discover a close association between FGFR3_∆7–9_ and the DNA demethylase Ten-Eleven Translocation-2 (TET2). Unlike other certain types of cancer, mutation of TET2 is rare in HCC. However, activation of FGFR3_∆7–9_ by FGF1 dramatically shortens TET2 half-life. FGFR3_∆7–9_, but not wild-type FGFR3, directly interacts with TET2 and phosphorylates TET2 at Y1902 site, leading to the ubiquitination and proteasome-mediated degradation of TET2. Overexpression of a phospho-deficient mutant TET2 (Y1902F) significantly reduces the oncogenic potential of FGFR3_∆7–9_ in vitro and in vivo. Furthermore, FGFR3_∆7–9_ significantly enhances HCC cell proliferation through the TET2-PTEN-AKT pathway. Specifically, TET2 offsets the elevation of p-AKT level induced by FGFR3_∆7–9_ through directly binding to PTEN promoter and increasing 5-hmC. Therefore, through phosphorylation and inhibition of TET2, FGFR3_∆7–9_ reduces PTEN expression and substantiates AKT activation to stimulate HCC proliferation. Together, this study identifies TET2 as a key regulator of the oncogenic role of FGFR3_∆7–9_ in HCC carcinogenesis and sheds light on new therapeutic strategies for HCC treatment.

## Introduction

Liver cancer is one of the most common cancers, and is the fourth lethal malignancy worldwide^1^. Moreover, the incidence of liver cancer continues to increase by 2 to 3% annually during 2007–2016 in United States^2^. Both genetic and epigenetic alterations play critical roles in HCC. In our previous study, we demonstrated that overexpression of fibroblast growth factor receptor 3 (FGFR3) plays an important role in HCC development^3^. Moreover, we have shown that FGFR3_∆7–9,_ a novel mutant transcript, links exon 6 to exon 10 directly and promotes the proliferation, migration, and metastasis of HCC cells both in vitro and in vivo^4^.

DNA methylation, which occurs mainly on the 5th carbon atom of cytosine (5-methylcytosine, 5-mC) in the context of CpG islands, has an important role in normal development and tumor development^5^. DNA methylation and demethylation are important for regulating chromosome structure, as well as gene expression. Ten-Eleven Translocation-2 (TET2) is a member of the dioxygenase family, which converts 5-mC to 5-hydroxymethylcytosine (5-hmC) and leads to demethylation at CpG islands^6^. TET2 is one of the most frequently mutated genes in hematopoietic malignancies, and its disruption is an early event in the onset of disease^7^. Patients carrying TET2 mutations often show significantly reduced global 5-hmC levels^8^. Decreased expression of TET proteins and lower 5-hmC levels are also general hallmarks of many solid cancer types, including liver, breast, lung, gastric, prostate, and breast cancer, as well as glioblastoma and melanoma^9–12^. Moreover, the decrease in 5-hmC levels can be attributed to the high proliferation rate of cancer cells^13^. However, the detailed regulation mechanisms of TET2 in HCC are unclear.

Searching for proteins that interact with and mediate the function of FGFR3_∆7–9_, we found that that FGFR3_∆7–9_ can significantly enhance proliferation in HCC cells through the TET2-PTEN-AKT signaling pathway. We demonstrated that TET2 was significantly downregulated in HCC, and lower TET2 expression levels were associated with poor prognosis. Moreover, FGFR3_∆7–9_ could directly interact with and phosphorylate TET2 at Y1902, which led to the ubiquitination and proteosomal degradation of TET2. TET2 downregulation by FGFR3_∆7–9_ attenuated PTEN expression, which strengthened AKT phosphorylation and promoted the proliferation and survival of HCC cells. The functional link between FGFR3_∆7–9_ and tumor suppressor TET2 revealed in our study may help develop new therapeutic strategies for HCC treatment.

## Results

### FGFR3_∆7–9_ interacts with TET2 and phosphorylates TET2 at Y1902

**Fig. 1:**
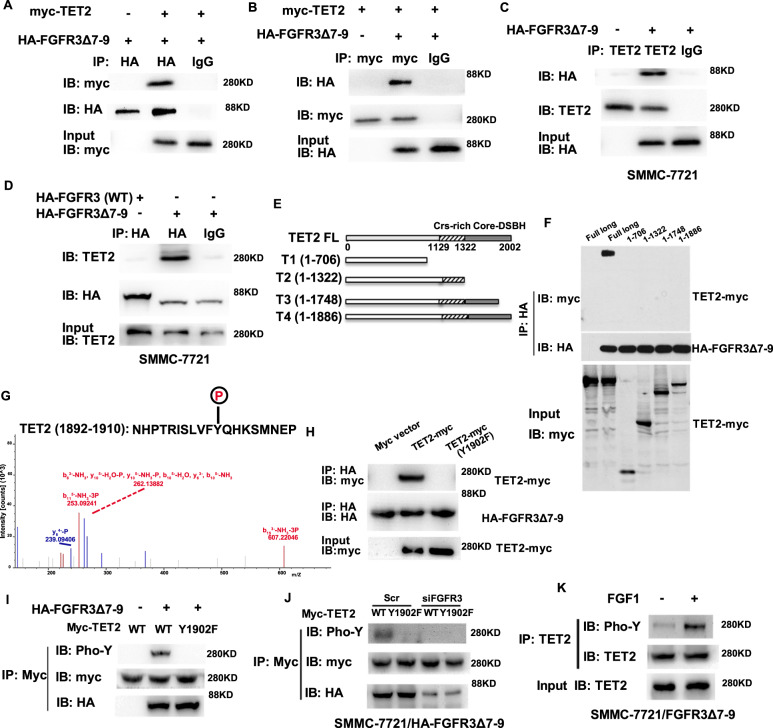
FGFR3_∆7–9_ phosphorylates TET2 at Y1902. **A**–**B** Immunoprecipitates (IP) from 293T cells transfected with HA-FGFR3_∆7–9_, myc-TET2, or both plasmids. **C**–**D** Immunoblot analysis of immunoprecipitation from SMMC-7721 cells stably expressing the HA-FGFR3_∆7–9_ construct. **E** The truncated versions of TET2, T1 (1–706 aa), T2 (1-1322), T3 (1-1748), and T4 (1-1886). **F** The interactions between FGFR3_∆7–9_ and truncated versions of TET2 were determined by immunoprecipitation. **G** The tyrosine phosphorylation site of TET2 was measured by Multistage mass spectrometry in SMMC-7721 cells stably expressing the HA-FGFR3_∆7–9_ construct; **H** SMMC-7721 cells were transfected with myc-WT-TET2 or myc-Y1902F-TET2, together with HA-FGFR3_∆7–9_. **I** TET2 tyrosine phosphorylation status was examined by immunoblot analysis after immunoprecipitation by anti-Myc (Myc-TET2). **J** TET2 phosphorylation status was detected in indicated cell lines. **K** TET2 phosphorylation status in SMMC-7721_FGFR3∆7–9_ cells in the presence and absence of FGF1.

Our previous study found that FGFR3_∆7–9_ has a stronger ability to promote the progression of HCC cells than wild type FGFR3^4^. TET2 was one of the potential downstream substrates of FGFR3_∆7–9_ by tandem mass spectrometry (data not shown). In a co-immunoprecipitation assay with ectopically expressed FGFR3_∆7–9_ and TET2, we found that HA-tagged FGFR3_∆7–9_ could precipitate myc-TET2 from transfected 293T cells (Fig. 1a). Conversely, myc-TET2 could also pull down His-FGFR3 (Fig. 1b). In addition, the physical interaction between TET2 and FGFR3_∆7–9_ was supported by experiments conducted in an HCC cell line (SMMC-7721) stably expressing FGFR3 (WT) or FGFR3_∆7–9_. Our results demonstrated that FGFR3_∆7–9_, but not wild type FGFR3, was able to co-immunoprecipitate with endogenous TET2 in a reciprocal manner (Fig. 1c, d).

To further identify the binding site of TET2 to FGFR3_∆7–9_, we first truncated TET2 into fragments T1, T2, T3, and T4 out of 2002 amino acids (Fig. 1e). All four fragments could not interact with FGFR3_∆7–9_, and only full length TET2 may mediate binding (Fig. 1f). Hence, the key binding site of TET2 to FGFR3_∆7–9_ was narrow-downed to1887–2002 fragment. Considering the distinctive role of FGFR3_∆7–9_ as a tyrosine kinase, TET2 may be tyrosine phosphorylated by FGFR3_∆7–9_ and we verified this hypothesis using a multistage mass spectrometry method. As expected, in this fragment, one solo tyrosine phosphorylation site of TET2 was identified at Y1902 (Fig. 1g). Then, we mutated TET2 Y1902 to Y1902F to pinpoint the speculated binding site to FGFR3_∆7–9_ of TET2. As expected, the Y1902F mutation of TET2 abolished the interaction between TET2 and FGFR3_∆7–9_ (Fig. 1h). More importantly, FGFR3_∆7–9_ could significantly increase the tyrosine phosphorylation of wild-type TET2 but not the Y1902F mutant (Fig. 1i). Hence, we confirmed Y1902 was the phosphorylated site of TET2 by FGFR3_∆7–9_. In addition, when FGFR3_∆7–9_ was silenced by siRNA, the tyrosine phosphorylation of TET2 by FGFR3_∆7–9_ could be apparently abolished (Fig. 1j). When FGF1, ligand of FGFR3, was further included in system, the tyrosine phosphorylation of TET2 could be increased consequently (Fig. 1k). Altogether, our findings indicated that Y1902 is the phosphorylation site of TET2 by FGFR3_∆7–9_.

### FGFR3_∆7–9_ negatively regulates TET2 stability and promotes its ubiquitination

**Fig. 2:**
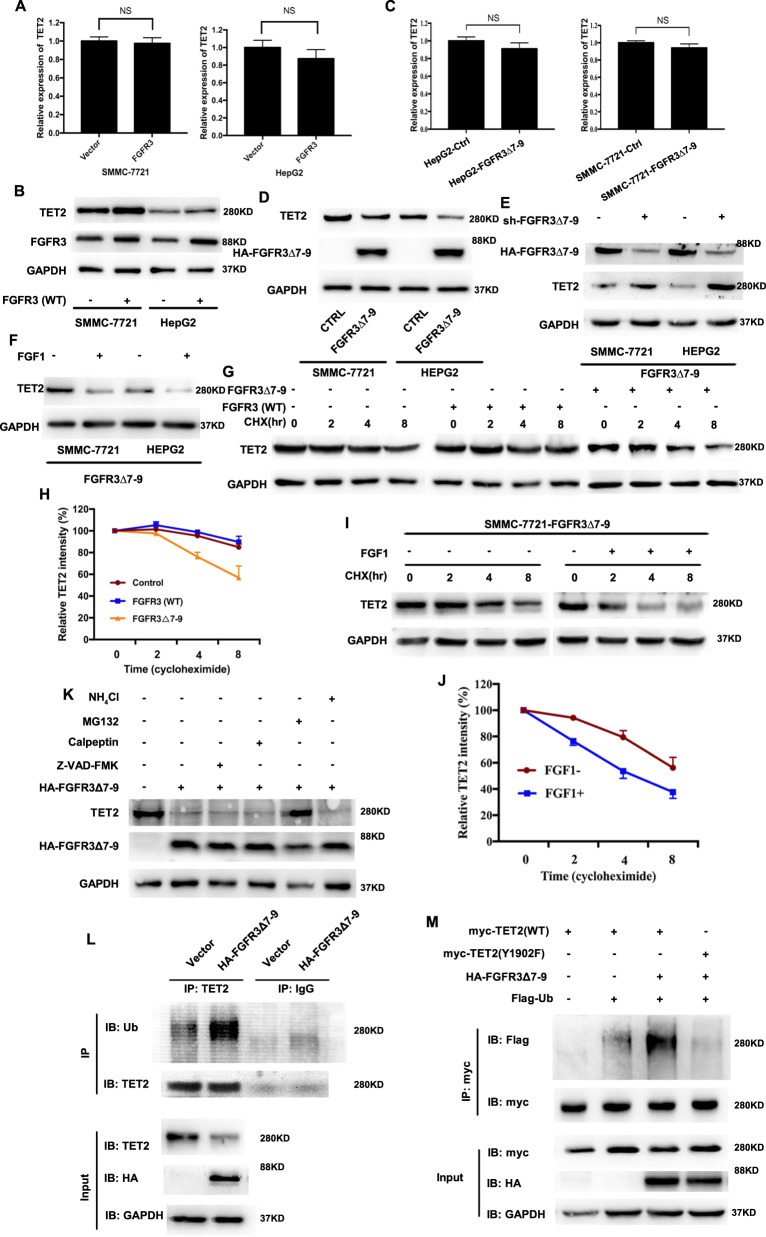
FGFR3_∆7–9_ negatively regulates TET2 stability. **A** qPCR analysis of TET2 expression in SMMC-7721 and HepG2 cells infected with empty vector or FGFR3-expressing plasmid. **B** Western blot analysis of TET2 expression in SMMC-7721 and HepG2 cells infected with empty vector or FGFR3-expressing plasmid. **C** qPCR analysis of TET2 expression in SMMC-7721 and HepG2 cells infected with empty vector or FGFR3_∆7–9_-expressing plasmid. **D** Western blot analysis of TET2 expression in SMMC-7721 and HepG2 cells infected with empty vector or HA-FGFR3_∆7–9_-expressing plasmid. **E** Western blot analysis of TET2 expression in SMMC-7721/FGFR3_∆7–9_ and HepG2/FGFR3_∆7–9_ after indicated treatment; **F** Western blot analysis of TET2 expression in SMMC-7721/FGFR3_∆7–9_ and HepG2/FGFR3_∆7–9_ in the presence and absence of FGF1 **G** SMMC-7721 cells were treated with 20 μg/ml CHX, and whole-cell lysates (WCL) were collected at the indicated time points for immunoblot analysis. **H** Semiquantification with GAPDH as a loading control. Relative TET2 levels at time 0 were set as 100%. **I** SMMC-7721/FGFR3_∆7–9_ cells were treated with 20 μg/ml CHX in the presence or absence of FGF1, and whole-cell lysates (WCL) were collected at the indicated time points for immunoblot analysis. **J** Semiquantification with GAPDH as a loading control. Relative TET2 levels at time 0 were set as 100%. **K** FGFR3_∆7–9_-mediated TET2 degradation is blocked by the proteasome inhibitor, MG132. SMMC-7721 cells were transfected with HA-FGFR3_∆7–9_ and then treated with inhibitors of lysosome (NH_4_Cl, 20 mM), calpain (Calpeptin, 50 μM), caspase (Z-VAD-FMK, 100 μM), or 26S proteasome (MG132, 10 μM) for 24 h, followed by immunoblotting analyses. **L** SMMC-7721/FGFR3_∆7–9_ and control cells were treated with MG-132 (10 μM for 24 h). Cell lysates were immunoprecipitated with antibodies against TET2 or IgG. Ubiquitination levels were analyzed by western blot. **M** Cells were transfected with indicated plasmids. TET2 ubiquitylation was examined by coupled IP-western.

The above results indicated the interaction between FGFR3_∆7–9_ and TET2. To further study the effect of FGFR3_∆7–9_ on TET2, we first determined whether FGFR3_∆7–9_ affected the expression of TET2. We examined the expression of TET2 at both the mRNA and protein levels by qPCR and immunoblotting, respectively, upon overexpression of FGFR3_∆7–9_ in SMMC-7721 and HepG2 cell lines. We found that both the mRNA and protein levels of TET2 were not altered by FGFR3 (Fig. 2a, b). However, although TET2 mRNA level was barely affected by FGFR3_∆7–9_, TET2 protein level was significantly decreased upon overexpression of FGFR3_∆7–9_ in both HCC cells (Fig. 2c, d). Meanwhile, downregulation of TET2 protein by FGFR3_∆7–9_ overexpression could be reversed by FGFR3_∆7–9_ knockdown (siFGFR3_∆7–9_) in Fig. 2e. On the other hand, activated by FGF1, FGFR3_∆7–9_ could further inhibit TET2 protein expression (Fig. 2f).

The disconnection between mRNA and protein levels of TET2 by FGFR3_∆7–9_ overexpression suggests post-translational regulation of TET2 by FGFR3_∆7–9_. To test this hypothesis, we performed a cycloheximide (CHX) chase experiment to determine TET2 half-life with or without FGF1 in SMMC-7721/FGFR3_∆7–9_ cell lines. We found FGFR3_∆7–9_, but not FGFR3 (wild type), could significantly shortened TET2 half-life (Fig. 2g, h). Furthermore, activation of FGFR3_∆7–9_ by FGF1 dramatically shortened TET2 half-life (Fig. 2i, j). These results suggested that FGFR3_∆7–9_, not wild type FGFR3, negatively regulated TET2 stability in HCC cells.

Based on above results, we used inhibitors of lysosome (NH_4_Cl), calpain (Calpeptin), and caspase (Z-VAD-FAK) to investigate potential mechanism. Results in Fig. 2K showed that none of these treatments could affect the accelerated TET2 degradation by FGFR3_∆7–9_. Instead, treatment with MG132, an inhibitor of the proteasome, effectively blocked TET2 half-life shortening by FGFR3_∆7–9_ activation, indicating that FGFR3_∆7–9_-mediated degradation of TET2 protein might be involved in the ubiquitin-proteasome pathway. Based on above data, we found that FGFR3_∆7–9_ promoted TET2 ubiquitylation (Fig. 2L). Given the possible phosphorylation site of TET2 by FGFR3_∆7–9_ in Y1902, we then found TET2 Y1902F mutation could abolish the ability of FGFR3_∆7–9_ to promote the ubiquitylation of TET2 (Fig. 2M). Together, these data strongly suggested that FGFR3_∆7–9_ could promote TET2 ubiquitin-proteasome degradation in a Y1902 phosphorylation-dependent manner.

### TET2 expression is downregulated in HCC tissues and predicts poor prognosis in HCC patients

**Fig. 3:**
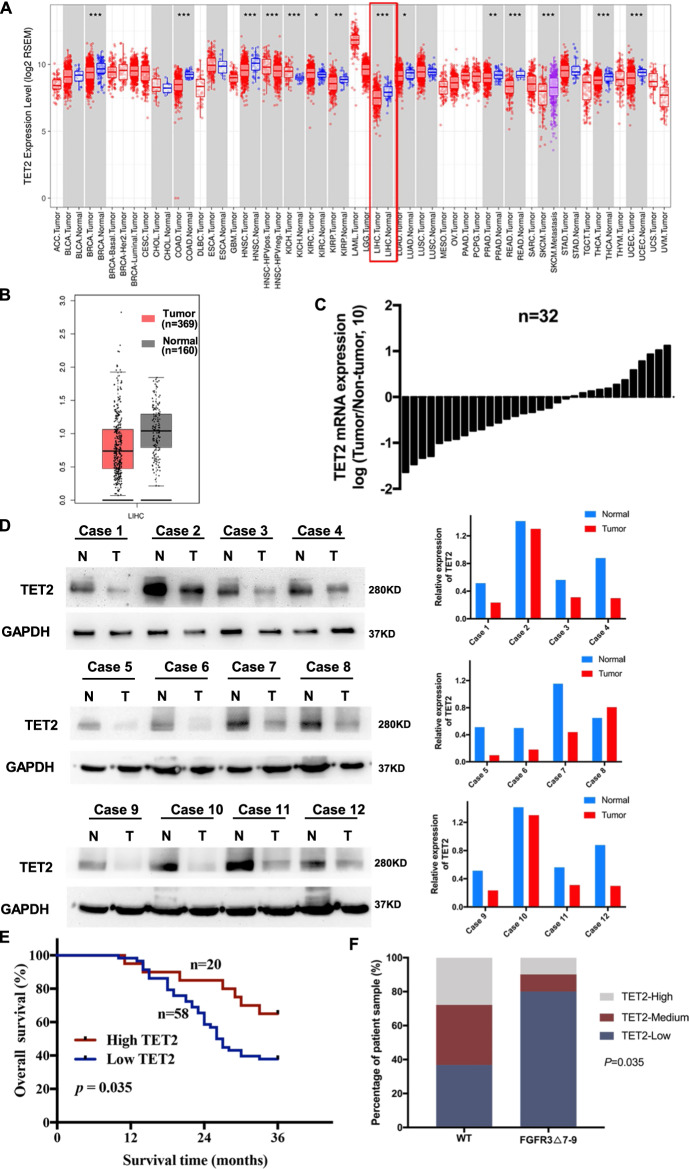
Low TET2 expression is associated with poor prognosis of HCC patients, and expression of TET2 is lower in FGFR3_∆7–9_ samples. **A** Downregulation of TET2 in several cancer types in the TCGA database. **B** The mRNA expression of TET2 in HCC is analyzed using TCGA data. **C** qRT-PCR analysis of TET2 mRNA expression in HCC and patient-matched normal tissues (*n* = 32). **D** Representative Western blot analysis of TET2 expression in 12 paired HCC samples. **E** Overall survival curves of the 78 patient cohort, with patients stratified based on TET2 expression level. **F** Statistical analysis of the correlation between the levels of TET2 and FGFR3_∆7–9_ (*P* = 0.035; *P* value was obtained using a Pearson *χ*2 test).

To explore the effects of downregulation of TET2 induced by FGFR3_∆7–9_, we firstly investigated the expression of TET2 and the relationship of FGFR3 in HCC. Based on the TCGA transcriptomic data, a number of cancer types, including HCC, we observed that lower TET2 expression levels in cancer tissues when compared with matched normal tissues (Fig. 3A, B). In our data panel, mRNA expression of TET2 was measured in 32 pairs of HCC and patient-matched normal tissues by quantitative RT-PCR (qRT-PCR). Consistent with the TCGA dataset, as shown in Fig. 3C, TET2 mRNA levels were significantly lower in HCC tissues than in normal tissues. Lower expression of TET2 in tumors was further confirmed at the protein level by western blot analyses (Fig. 3D). To investigate the role of TET2 in HCC progression, we determined the expression levels of TET2 in HCC clinical samples by immunohistochemistry (IHC) staining of tissue microarrays (TMAs), which contain 78 pairs of tumor and adjacent normal tissues. In our cases, positive expression of TET2 was detected in 20 (25.6%) of the tumor tissues, while only 50 (43.1%) of the adjacent normal specimens showed a positive signal (*P* = 0.009) (Table 1). From this, we confirmed that TET2 did be downregulated in HCC tissues in clinical scenario. Clinicopathological features analyses showed that decreased IHC signal of TET2 was correlated with larger tumor size (*P* = 0.036, Table 1). However, no significant correlation was observed between TET2 expression and other parameters, such as gender, age, TNM stage, distant metastasis, or relapse (*P* > 0.05, Table 1). Regarding the clinical prognosis, lower TET2 expression levels were associated with a worse 3-year overall survival (*P* = 0.035, Fig. 3E). IHC staining also indicated that the level of TET2 is lower in HCC samples with FGFR3_∆7–9_ (Fig. 3F, *P* = 0.035). Overall, our clinical data suggested that downregulation of TET2 is correlated with poor prognosis and the protein level of TET2 was downregulation in HCC with FGFR3_∆7–9_.

**Table 1:** Clinicopathological features and correlation of TET2 expression in HCC.

	Case number	TET2 expression		
Characteristics	*N* = 78	Low (58)	High (20)	*P*-value
Gender				0.536
Male	61	44	17	
Female	17	14	3	
Age				0.303
≤55	38	26	12	
>55	40	32	8	
Tumor diameter (cm)				0.036
≤5.0	41	26	15	
>5.0	37	32	5	
TNM stage				0.207
I + II	37	25	12	
III + IV	41	33	8	
Distant metastasis				1.000
No	65	48	17	
Yes	13	10	3	
Pathological stage				0.499
I + II	64	46	18	
III + IV	14	12	2	
Relapse				0.441
No	40	28	12	
Yes	38	30	8	

### Y1902 phosphorylation inhibits TET2 tumor suppressing function in HCC

**Fig. 4:**
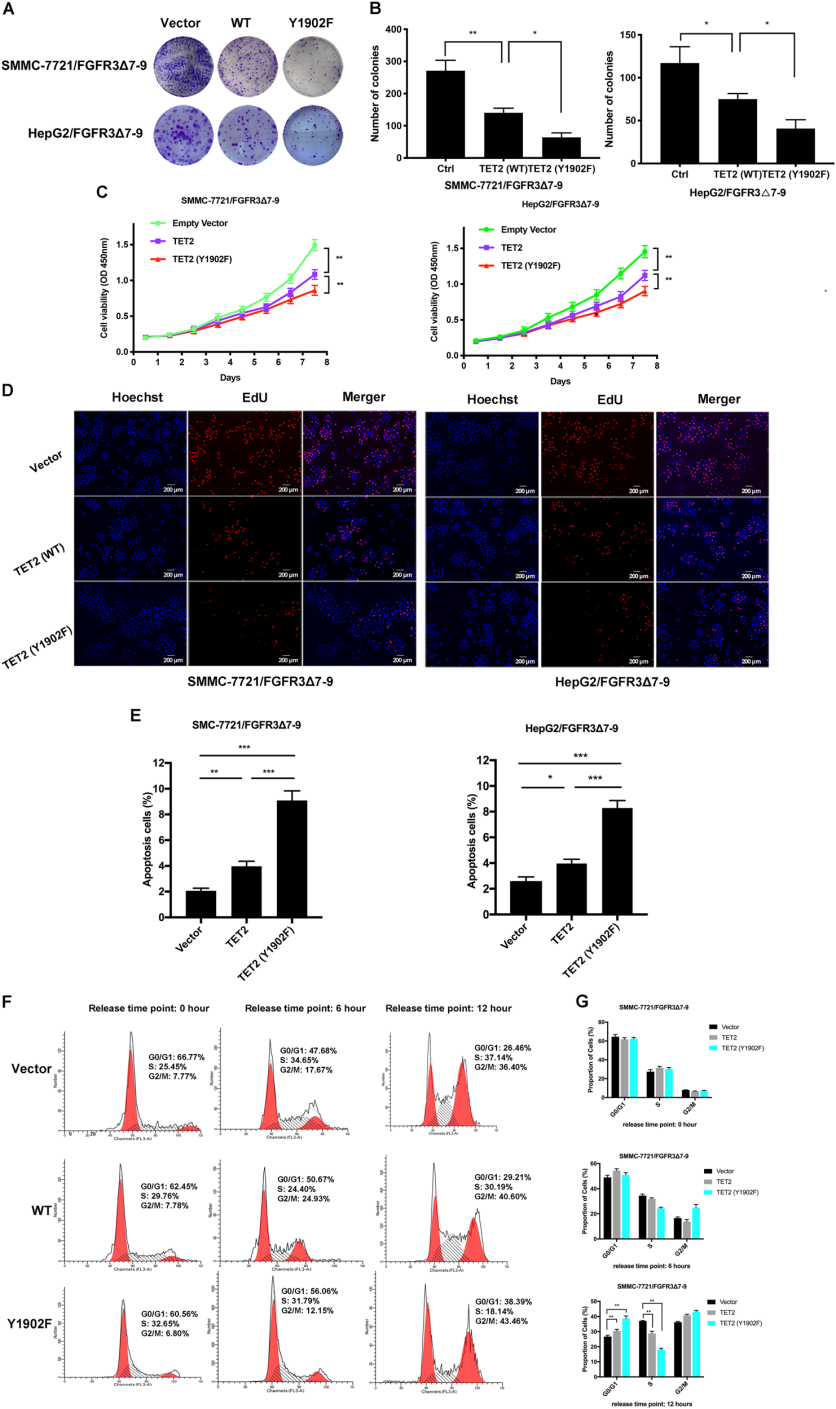
Phosphorylation at the Y1902 site of TET2 increases HCC cell proliferation in vitro. **A**–**B** Representative images of clone formation on plastic plates with SMMC-7721_FGFR3∆7–9_ and HepG2_FGFR3∆7–9_ cell lines expressing TET2 (wild type and Y1902F mutant) or empty vector. Quantification of cell growth is shown in **B** (*n* = 3, independent *t*-test, *P < 0.05, **P < 0.01). **C** CCK-8 cell proliferation assay for SMMC-7721_FGFR3∆7–9_/HepG2_FGFR3∆7–9_ cells that stably express TET2 (WT) or TET2 (Y1902F). Cells transfected with empty vector were used as a control. **D** Cell proliferation of indicated sublines, measured by EdU incorporation (40×). **E** The ratio of apoptotic cells in indicated sublines. *P < 0.05, **P < 0.01. **F–G** Indicated sublines were serum-deprived (starved) for 24 hours and then maintained in culture medium with 10% FBS (released) for 0, 6 or 12 h. **P < 0.01.

In our previous study, FGFR3_∆7–9_ showed more strong ability to promote HCC cell proliferation than wild type FGFR3^4^. Given the critical role of Y1902 phosphorylation by FGFR3_∆7–9_ in controlling TET2 stability, we continued to assess the role of this phosphorylation for the function of FGFR3_∆7–9_. First, we transfected wild-type TET2 or the Y1902F phospho-deficient mutant into SMMC-7721/FGFR3_∆7–9_ or HepG2/FGFR3_∆7–9_. In an anchorage-dependent growth assay, TET2 (Y1902F) formed fewer and smaller colonies compared with its mock and TET2 (WT) cunterparts (Fig. 4A, B). Moreover, TET2 (Y1902F) also displayed much lower proliferation potential than the SMMC-7721/FGFR3_∆7–9_ or HepG2/FGFR3_∆7–9_ control and TET2 (WT) cells (Fig. 4C). Furthermore, Edu staining also confirmed the effects of TET2 Y1902 phosphorylation on proliferation of SMMC-7721/FGFR3_∆7–9_ and HepG2/FGFR3_∆7–9_ cells (Fig. 4D). Meanwhile, as assessed by flow cytometry, TET2 (Y1902F) drastically induced apoptosis compared to control and TET2 (WT) cells (Fig. 4E). In addition, cell cycle analysis revealed that TET2 (Y1902F) inhibited the G1-S phase transition (Fig. 4F, G). Altogether, TET2 (Y1902F) demonstrated more apparent inhibitive effect on proliferation than TET2 (WT) in SMMC-7721/FGFR3_∆7–9_ and HepG2/FGFR3_∆7–9_ cells HCC cells.

### FGFR3_∆7–9_ decreases the level of PTEN and TET2 restores PTEN expression

PTEN is critical for inhibiting cancer cell migration, invasion and proliferation. We observed lower PTEN expression levels in HCC tumor tissues versus corresponding normal tissues in the TCGA data, and low PTEN mRNA levels were associated with a worse overall survival (Supplementary Fig. S1A and S1B). Moreover, lower PTEN protein levels also showed a worse disease free survival and overall survival (analyzed through the TRGAted: https://nborcherding.shinyapps.io/TRGAted/) (Supplementary Fig. S1C and S1D). PTEN loss is inversely correlated with constitutive activation of the PI3K/AKT signaling pathway^14^, indicating PTEN inhibits the activation of the PI3K/AKT pathway.

**Fig. 5:**
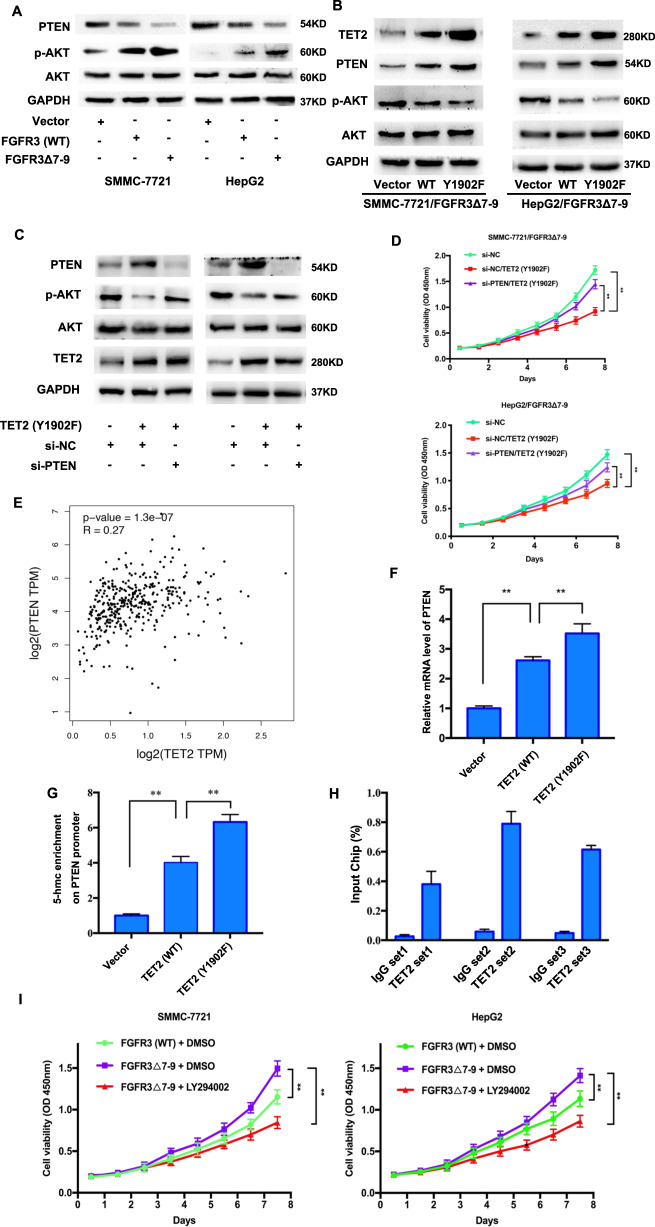
Phosphorylation at TET2 Y1902 results in decreased PTEN expression. **A** The levels of PTEN, AKT, and p-AKT were measured by immunoblotting in SMMC-7721 and HepG2 cells expressing FGFR3 (WT) or FGFR3_∆7–9_. **B** Western blot analysis for TET2, PTEN, AKT, and p-AKT in SMMC-7721_FGFR3∆7–9_ and HepG2_FGFR3∆7–9_ cell lines expressing TET2 (wild type and Y1902F mutant) or empty vector. **C** Western blot analysis for PTEN, AKT, and p-AKT in SMMC-7721_FGFR3∆7–9_ and HepG2_FGFR3∆7–9_ with indicated treatment. **D** CCK-8 cell proliferation assay for indicated cell lines. **E** The correlation of TET2 and PTEN from TCGA data analysis (*R* = 0.27, *P* < 0.001). **F** The mRNA levels of PTEN were measured by qRT-PCR in SMMC-7721_FGFR3∆7–9_ and HepG2_FGFR3∆7–9_ cell lines expressing TET2 (wild type and Y1902F mutant) or empty vector. **G** The 5-hmC content at the PTEN promoter in SMMC-7721_FGFR3∆7–9_ cell lines expressing TET2 (wild type and Y1902F mutant) or empty vector, **P < 0.01. **H** ChIP assay was performed to determine the interaction between TET2 and the PTEN promoter. **P < 0.01. **I** Inhibition of signaling downstream of PTEN decreases cell proliferation induced by FGFR3_∆7–9._ CCK-8 cell proliferation assay for cell treated with DMSO and LY294002.

Overexpression of FGFR3 significantly downregulated the expression of PTEN and increased the level of p-AKT, and these effects were more remarkable in FGFR3_∆7–9_ than in wild type FGFR3 (Fig. 5a). Meanwhile knockdown TET2 further increased the level of p-AKT and decrease the level of PTEN in present of FGFR3_∆7–9_ (Supplementary Fig. S2). In Fig. 5b, we found that TET2 (Y1902F) could significantly increase PTEN levels and decrease p-AKT levels. It revealed that it was TET2 (Y1902F) that could affect the downregulated of PTEN and upregulation of p-AKT induced by FGFR3_∆7–9_.

Based on that, in Fig. 5c, we knocked down PTEN in SMMC-7721/FGFR3_∆7–9_ and HepG2/FGFR3_∆7–9_ cells with TET2 (Y1902F). The results showed that PTEN knockdown could increase p-AKT (Fig. 5c), and promote HCC cell proliferation (Fig. 5d). It indicated it was PTEN that downregulated p-AKT and inhibited HCC cell proliferation induced by TET2 (Y1902F).

### TET2 upregulates PTEN expression via increasing 5-hmC level in PTEN promoter area

From above, we found that TET2 (Y1902F) dramatically increased PTEN levels, and we then explored the possible underlining mechanisms. Based on the TCGA database, we found that PTEN methylation level is higher in tumor than normal tissues (Supplementary Fig. S3A), and PTEN methylation level is negative related to PTEN expression (Supplementary Fig. S3B). Moreover, the methylation level of PTEN is associated with poor prognosis (Supplementary Fig. S3C). Therefore, regulation of methylation level of PTEN is one of mechanisms for altering the expression of PTEN in HCC. Furthermore, TCGA data analyses demonstrated that PTEN was positively correlated with TET2 (Fig. 5e), and TET2 (Y1902F) could significantly increase PTEN mRNA levels (Fig. 5F). TET2’s function was made possible by regulating its targets’ 5-hmC level in promoter area, and PTEN is canonically modulated by promoter methylation status. Consequently, we analyzed the 5-hmC content in the promoter region of PTEN. Evidenced by quantification of 5-hmC levels in genomic DNA by methylation-sensitive qPCR, we found that knockdown of TET2 decrease the level of 5-hmc in PTEN promoter region (Supplementary Fig. S4A). Furthermore, we found that TET2 (Y1902F) induced much higher levels of 5-hmC in PTEN promoter region compared with its mock and TET2 (WT) counterparts (Fig. 5g). Meanwhile, the PTEN promotor methylation level was lowest in the TET2 (Y1902F) group (Supplementary Fig. S4B). Furthermore, the results from chromatin immunoprecipitation (ChIP) assay definitely confirmed the direct binding of TET2 to the PTEN promoter using three sets of primers (Fig. 5h). Moreover, we found cell proliferation increased by FGFR3_∆7–9_ could be inhibited by LY294002, an inhibition of PI3K/AKT pathway (Fig. 5i). We also used Wortmannin, a specific and irreversible inhibitor for PI3K/AKT, and found the consist result with LY294002 (Supplementary Fig. S5). In all, our results indicated that TET2 could directly bind to the PTEN promoter, then increase its 5-hmC level and boost PTEN transcription consequently.

### Y1902 phosphorylation inhibits TET2 tumor suppressor function in HCC in vivo

**Fig. 6:**
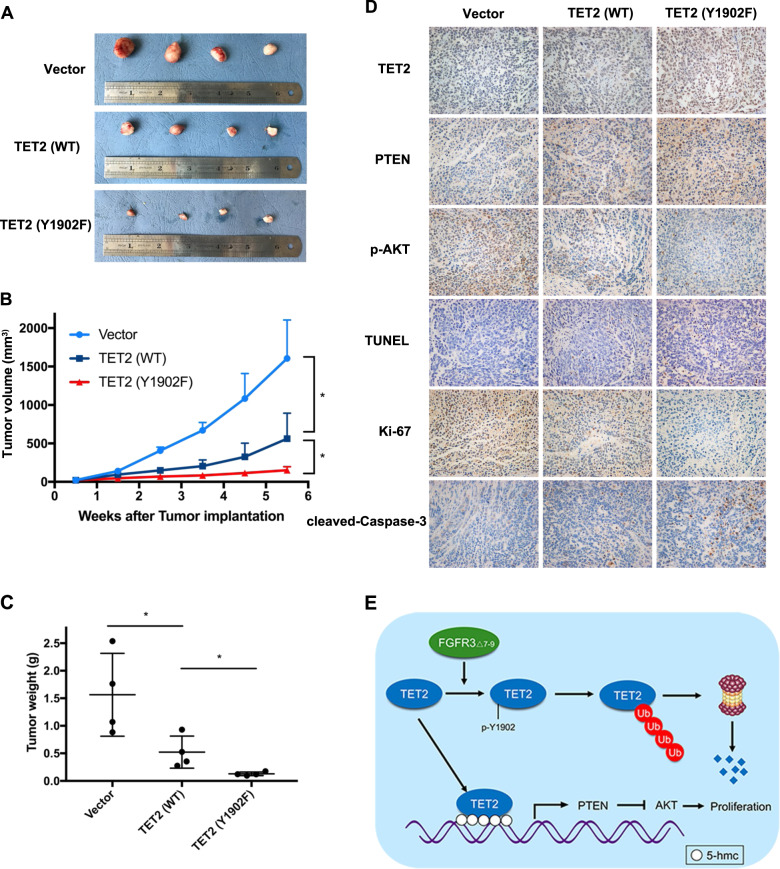
Phosphorylation at the Y1902 site inhibits TET2 tumor suppressing function in HCC in vivo. **A**–**C** Images and quantification (tumor volume at indicated time points, tumor weight at endpoint) of subcutaneous tumors formed by SMMC-7721_FGFR3∆7–9_/vector, SMMC-7721_FGFR3∆7–9_/TET2 (WT) and 7721_FGFR3∆7–9_/TET2 (Y1902F). *P < 0.05. **D** Representative images of IHC (TET2, PTEN, p-AKT, Ki-67 and cleaved-caspase 3) and TUNEL assay of subcutaneous tumors formed by SMMC-7721_FGFR3∆7–9_/vector, SMMC-7721_FGFR3∆7–9_/TET2 (WT) and SMMC-7721_FGFR3∆7–9_/TET2 (Y1902F) (200×). **E** Schematic of FGFR3_∆7–9_ regulation of TET2 stability to influence its tumor suppressive functions in hepatocellular carcinoma.

For in vivo confirmation of phenotype of TET2 (Y1902F), a recombinant lentivirus harboring TET2 (Y1902F) and TET2 (WT) was transfected into SMMC-7721/FGFR3_∆7–9_ cells. The stable clone expressing ectopic TET2 (Y1902F) and TET2 (WT) was subcutaneously injected into the flank of each athymic nude mouse, and an equal volume of cells transfected with the empty vector was injected into the opposite flank of the same mouse as the negative control. As shown in Fig. 6a, b, TET2 (WT) cells caused smaller tumor masses than the mock vector control after 6 weeks of observation (SMMC-7721/FGFR3_∆7–9_/TET2 (WT), 562.8 ± 332.15 mm^3^; SMMC-7721/FGFR3_∆7–9_/vector, 1604.6 ± 500.2 mm^3^; *P* < 0.05). Moreover, the tumors of TET2 (Y1902F) overexpressing cells were significantly smaller than those in TET2 (WT) group (SMMC-7721/FGFR3_∆7–9_/TET2 (Y1902F), 151.7 ± 46.7 mm^3^; *P* < 0.05). Meanwhile, tumors derived from the offspring subclones with TET2 (Y1902F)-overexpressing cells were significantly lighter than those in the TET2 (WT) and control group (TET2 (Y1902F), 0.131 ± 0.033 g; TET2 (WT), 0.517 ± 0.291 g; vector, 1.564 ± 0.752; *P* < 0.05) (Fig. 6c). Furthermore, tumor sections from the nude mouse model were immunohistochemically stained for TET2, PTEN, and p-AKT. We observed that TET2 and PTEN expression was increased in the TET2 (Y1902F) group compared with the TET2 (WT) and vector group (Fig. 6d). However, p-AKT expression was decreased in the TET2 (Y1902F) group compared with other groups. Proliferation was assessed using IHC of Ki67, which showed reduced numbers of positively stained cells in TET2 (Y1902F) tumors compared to TET2 (WT) and vector tumors. Apoptotic cells detected by TUNEL assay and IHC of cleaved-caspase 3, apoptosis was increased in TET2 (Y1902F) group compared to other groups.

## Discussion

FGFR family of proteins have an extracellular ligand-binding domain, a transmembrane domain, and a split intracellular kinase domain^15^. Members of the FGFR family can bind to FGFs, resulting in receptor dimerization, autophosphorylation, and downstream signal transduction^16^. Investigations demonstrated that FGFRs might play important roles in a variety of processes, including cellular proliferation, differentiation, and angiogenesis^17–19^. In our previous study, we found that, among FGFR family members, only FGFR3 was elevated in HCC specimens, which is positively correlated with poor clinicopathologic parameters^3^.

Genome Instability and Mutation is one of the hallmarks of Cancer^20^. Activating mutations of FGFR3 play important roles in the progression of chondrosarcoma, superficial bladder tumors and urothelial cell carcinomas^21^. Several novel mutant transcripts caused by aberrant splicing and activation of cryptic splice sequences have been reported in digestive tract tumors^22^. In our previous study, we identified a novel transmembrane mutation of FGFR3, FGFR3_∆7–9_, which lacks exons encoding the immunoglobulin-like III domain related to bind FGFs. FGFR3_∆7–9_ showed a much stronger affinity to FGFs than wild-type FGFR3, and promoted the proliferation, migration, and metastasis of HCC cells both in vitro and in vivo^4^.

FGFR3_∆7–9_ performs aberrant ligand binding ability and self-activity independent FGF1 stimulation. Besides, FGFR3_∆7–9_ also shows aberrant binding and phosphorylating ability. We further identified an FGFR3_∆7–9_’s aberrant binding protein, TET2. Emerging evidence suggests that TET families may play unique roles in many biological processes, such as gene control mechanisms, DNA methylation, and involvement in many diseases, especially cancer. As a tumor suppressor, loss of function of TET2 caused by mutation or deletion has previously been reported in several types of cancer^7,23–25^. Although loss of 5-hmC has been observed in various cancers^9,26,27^, TET2 mutations are only frequently detected in leukemia. Using cBio Cancer Genomics Portal (cBioPortal) databases, we also found that mutation of TET2 was frequent in cancers, such as uterine cancer, acute myeloid leukemia, and melanoma (Supplementary Fig. S6A). However, the data from 373 individual HCC patients in the TCGA dataset showed that <1% of specimens had TET2 mutations (3 out 373) (Supplementary Fig. S6B). Therefore, unlike other certain type of cancer, mutation of TET2 was a rare event in HCC. IDH1/2 mutations resulting in production of oncometabolite D-2-HG inhibits TETs and many other epigenetic enzymes, but are also highly restricted to gliomas and leukemia^28,29^. Our data now suggest altered post-translational modification as another mechanism for deregulating TET2 functions in HCC. Several pathways regulating TETs protein stability have been reported, such as the caspase^30^ and calpains^31^ pathways. Our data show that TET2 stability could also be regulated by the ubiquitin-proteasome pathway in HCC. FGFR3_∆7–9_ significantly reduced the protein level of TET2, rather than affected the mRNA level of TET2, and we found it was the ubiquitin-proteasome pathway that got involved in FGFR3_∆7–9_-mediated TET2 degradation. Furthermore, we found that FGFR3_∆7–9_, but not wild-type FGFR3, could directly interact with TET2 and phosphorylate the Y1902 site to destabilize TET2.

Aberrant promoter hypermethylation leading to inappropriate transcriptional silencing of tumor suppressor genes is often found in various human neoplasms, including HCC, colorectal and gastric cancers^32–34^. TET2 is one member of the TET family that enzymatically converts 5‐methylcytosine to 5‐hmC, which is an intermediate of DNA demethylation^35^. The results presented here demonstrate that TET2 is an inhibitor of FGFR3_∆7–9_. Meanwhile, TET2-Y1902F mutation exhibited a more remarkable inhibition of FGFR3_∆7–9_. Oncogenic FGFR3 signaling occurs through the PI3K/AKT, Ras/Raf/MEK/ERK, and JAK/STAT pathways^36–38^. FGFR3_∆7–9_ more potently induced phosphorylation of the ERK and AKT kinases, leading to abnormal downstream signaling through the ERK and PI3K/AKT/mTOR pathways. In this study, we found that FGFR3_∆7–9_ could significantly activate the PI3K/AKT and ERK pathways, and inhibited the expression of PTEN. However, TET2 could promote the re-expression of PTEN. Moreover, TET2 could reduce the elevated p-AKT induced by FGFR3_∆7–9_, which depended on the upregulation of PTEN. We further confirmed that TET2 could directly bind to the PTEN promoter and increase 5-hmC. Therefore, through the phosphorylation and inhibition of TET2, FGFR3_∆7–9_ reduced PTEN expression, and then rescued the level of p-AKT to trigger the cell growth advantage.

In summary, by exploring the signaling pathways unique to HCC and absent from other types of cancer, we demonstrated, for the first time, that FGFR3_∆7–9_ phosphorylates the tumor suppressor TET2 at Y1902, which promotes TET2 ubiquitination and destruction. Thus, the dysregulation of TET2 targets, such as PTEN, promotes HCC progression. These findings have revealed important aspects of the functional roles of oncogenic FGFR3_∆7–9_ signaling in HCC and may contribute to designing new therapeutic strategies to treat hepatic cancer.

## Materials and methods

### Patients, tissues specimens, and cell culture

Seventy-eight sets of HCC tissues and adjacent normal liver tissues (confirmed by pathology) were collected from patients who underwent curative surgery in Ruijin Hospital Shanghai Jiao Tong University School of Medicine from Jan. 2014 to Dec. 2015. Moreover, another two cohorts collected between 2016 and 2017 in accordance with the same protocol were also used in this study for qRT-PCR or western blot. Written informed consent was obtained and the research was approved by the local Ethics Committee and the Institutional Review Board. All patients were fully informed of the experimental procedures and were made aware of the potential risks and complications of the proposed treatment scheme. Patient data including demo-graphics, operative procedures, pathology, and complications, were prospectively collected. The human HCC cell lines SMMC-7721 was obtained from Shanghai Institute of Biochemistry and Cell Biology, Chinese Academy of Sciences. HepG2 and human embryonic kidney cell 293T were purchased from ATCC. HepG2 cells were grown in Eagle’s minimum essential medium, SMMC-7721 cells were cultured in RPMI 1640 medium and 293T cells were maintained in DMEM. Culture medium was added 10% FBS, 100 IU/ml penicillin and 100 IU/ml streptomycin.

### Immunohistochemistry

Briefly, paraffin-embedded tissue sections were dewaxed in xylene, re-hydrated in an ethanol series (100–50%) and treated in citrate buffer (pH 6.0) for antigen retrieval. After inhibition of endogenous peroxidase activity for 20 min with methanol containing 3% H_2_O_2_, slides were then stained with anti-TET2 (ABclonal Technology, A5682), PTEN (Cell Signaling Technology, #9599), anti-p-AKT (Cell Signaling Technology, #4060), anti-Ki-67 (Abcam, ab16667), or anti-cleaved-caspase 3 (Cell Signaling Technology, #9664) antibody at 4 °C overnight. The next day, slides were incubated in biotinylated secondary antibody for 30 min at 37 °C, visualized with DAB solution and counterstained with hematoxylin. Pictures were taken under a light microscope. The staining intensity was graded in four segments on a 3-point scale (staining scores): no staining (0 points), light brown staining (1 point), brown staining (2 points) and dark brown staining (3 points). The number of positive cells was divided into four grades (percentage scores): <5% (0), 5 – 30% (1), 31 – 70% (2), and 71 – 100% (3). TET2 staining was calculated by the following formula: overall staining score = intensity score × percentage score. A final score ≤3 was defined as negative staining, and >3 as positive staining.

### RNA isolation and quantitative real-time PCR

Total RNA was extracted using Trizol reagent (ThermoFisher, 15596026) and complementary DNA was synthesized with the Reverse Transcription system (Toyobo, FSQ-101) according to the manufacturer’s instructions. Real-time PCR was performed using SYBR Green PCR Master Mix (ThermoFisher, 4367659). The primers used for amplification are listed in Supplementary Table S1.

### Enzymatic chromatin immunoprecipitation assay

Enzymatic chromatin immunoprecipitation (ChIP) assays were performed using the Enzymatic ChIP Kit (Cell Signaling Technology, #9005) according to the manual. Briefly, HCC cells were cross-linked in 1% formaldehyde solution for 10 min at room temperature, followed by the addition of 125 mM of glycine for 5 min. Antibodies including anti-TET2 (Cell Signaling Technology, #18950) and normal IgG were used for each immunoprecipitation. Immunoprecipitated and input DNAs were subjected to qRT-PCR analyses. The primers used for amplification are listed in Supplementary Table S1.

### Immunoprecipitation and immunoblot analyses

Immunoprecipitation analyses were performed using the Direct Magnetic IP/Co-IP Kit (ThermoFisher, 88828) according to the manual. Briefly, indicated antibodies were bound to the beads for 60 min. Cell lysates were incubated with antibody-bound beads overnight at 4 °C. Washed the beads twice with Wash Buffer and once with ultrapure water. Eluted bound antigen.

For Western blot analyses, the cells were digested in RIPA buffer with presence of Protease Inhibitor Cocktail (ThermoFisher, 87785). Protein concentration was quantified using the BCA Protein Assay Kit (ThermoFisher, 23227). After electrophoresis in SDS-PAGE, the proteins were transferred to PVDF membranes (Bio-Rad, 1620177). After blocking with PBS containing 5% nonfat milk, blots were immunoblotted with the indicated primary antibodies. The antibodies included anti-HA (Cell Signaling technology, #3724) and anti-myc (Cell Signaling technology, 2276), anti-FGFR3 (Cell Signaling technology, #4574), anti-PTEN (Cell Signaling Technology, #9599), anti-Akt (Cell Signaling Technology, #4691), anti-p-Akt (Cell Signaling Technology, #4060), anti-TET2 (Cell Signaling Technology, #18950) and anti-GAPDH (Cell Signaling technology, #5174).

### Plasmids construction and transfection

Plasmid construction was described previously^4^. Plasmids were synthesized by Genechem (Genechem Co. Ltd., Shanghai, China) and were transfected using Lipofectamine 2000 reagent (Invitrogen) following the manufacturer’s protocol. SMMC-7721or HepG2 was transfected by FGFR3_∆7–9_ to establish stable colony of SMMC-7721/FGFR3_∆7–9_ or HepG2/FGFR3_∆7–9_ which overexpressed FGFR3_∆7–9_ in SMMC-7721 or HepG2. Wild type TET2 or Y1902F mutant TET2 were then transfected into SMMC-7721/FGFR3_∆7–9_ or HepG2/FGFR3_∆7–9_, overexpressing TET2 or TET2 Y1902F, respectively. The primers designed for constructing TET2 truncated fragments were as follows: Forward-GACGATATC ATGGAACAGGATAGAACCAAC; Reverse (706)-GACGCGGCCGCTCACTGTTGATTCAAGTGCTGTTT; Reverse (1322)-GACGCGGCCGC TCA CAGTTTCTCTTCCTCTTTTGG; Reverse (1748)-GAC GCGGCCGC TCA GTTTGGATTGCTCAGATTGGG; Reverse (1886)-GACGCGGCCGC TCA CTCCCGTTTCACTTTTTTGCC.

### Cell viability and colony formation assay

The Cell Counting Kit-8 (CCK-8, Dojindo, Kumamoto, Japan) was used for the cell proliferation assay. In brief, cells were plated in 96-well plates at 2000 cells per well (four biological replicates) and incubated at 37 °C with 5% CO_2_. Cell viability was quantified by measuring OD450 every 24 h using a microplate reader (Epoch; BioTek, Winooski, VT). For the colony formation assay, 1000 cells were plated per well in 6-well plates and cultured at 37 °C with 5% CO_2_ for 14 days. Colony formation was detected by staining with 0.1% crystal violet in methanol for 30 min. Data were obtained from three independent experiments.

### Apoptosis detection and cell cycle analyses

Flow cytometric assays of apoptosis and cell cycle were performed as previously described^39^. Briefly, both attached and floating cells were harvested, washed twice with ice-cold PBS and suspended in 100 µl binding buffer. Cells were incubated with 3 µl FITC-Annexin V and 5 µl PI at room temperature for 15 min in the dark. Next, an additional 300 μl 1× Binding Buffer was added to each sample, and then apoptosis was analyzed by flow cytometry (FACSCalibur; Becton Dickinson, Sparks, MD) according to the manufacturer’s instructions. For cell cycle analysis, single-cell suspensions were fixed with 70% cold ethanol at 4 °C overnight. Afterwards, samples were washed twice with cold PBS, incubated with PI (50 µg/ml) at 37 °C for 30 min in the dark, and then analyzed by flow cytometry (FACSCalibur).

### EdU staining

An EdU dye assay was performed using the Cell-Light EdU Apollo 567 In Vitro Imaging Kit (RiboBio Technology, Guangzhou, China) according to the manufacturer’s instructions. The labeled cells were counted under a fluorescence microscope. The experiments were independently repeated in triplicates.

### Quantification of 5-hmC levels in genomic DNA by methylation-sensitive qPCR

Genomic DNA was incubated with T4 Phage β‐glucosyltransferase (New England Biolabs, Ipswich, MA) by following the manufacturer’s protocol. First, 100 ng of glucosylated genomic DNA was digested with *Msp*I, or without enzyme (mock) at 37 °C overnight and then incubated for 20 min at 80 °C for enzyme deactivation. *Hpa*II‐resistant or *Msp*I‐resistant DNA fraction was quantified by qPCR and normalizing to the mock control. *Msp*I‐resistant DNA represents the 5hmC DNA fraction, whereas the fraction of 5-mC DNA was calculated by subtracting the 5-hmC fraction from the resistance to *Hpa*II. Primers were listed in Supplementary Table S1.

### Xenograft tumor model

HCC cell xenograft models in mice were established as previously described^4^. To establish the xenograft tumor model, HCC cells were injected into right frank of nude mice for in vivo tumor growth assay. SMMC-7721_FGFR3∆7–9_/Vector, SMMC-7721_FGFR3∆7–9_/TET2, and SMMC-7721_FGFR3∆7–9_/TET2 Y1902F cells (1 × 10^6^ cells) were subcutaneously injected into 4-week-old male BALB/c nude mice (Institute of Zoology, Chinese Academy of Sciences). Each group included 4 mice. Tumor nodules were measured and were calculated using the following formula: tumor volume = (width^2^ × length)/2. Mice were euthanized 6 weeks after injection. All animal studies were approved by the Ethics Committee of Ruijin Hospital, Shanghai Jiao Tong University School of Medicine.

### Statistical analyses

An ANOVA and Student’s *t* test were used for comparison among groups. The Mann–Whitney U test was used for comparison of tumor volume. Categorical data was evaluated with a c2 test or Fisher exact test. The survival curve was plotted using the Kaplan–Meier method and compared by the log-rank test. A *P* value less than 0.05 was considered to be significant.

## Supplementary information

Supplementary Figure Legends

Supplementary Figure 1

Supplementary Figure 2

Supplementary Figure 3

Supplementary Figure 4

Supplementary Figure 5

Supplementary Figure 6

Supplementary Table S1.

## References

[1] Bray F (2018). Global cancer statistics 2018: GLOBOCAN estimates of incidence and mortality worldwide for 36 cancers in 185 countries. CA Cancer J. Clin..

[2] Siegel RL, Miller KD, Jemal A (2020). Cancer statistics, 2020. CA Cancer J. Clin..

[3] Qiu WH (2005). Over-expression of fibroblast growth factor receptor 3 in human hepatocellular carcinoma. World J. Gastroenterol..

[4] Li K (2016). Phenotypic and signaling consequences of a novel aberrantly spliced transcript FGF receptor-3 in hepatocellular carcinoma. Cancer Res..

[5] Jones PA, Baylin SB (2007). The epigenomics of cancer. Cell.

[6] Rasmussen KD, Helin K (2016). Role of TET enzymes in DNA methylation, development, and cancer. Genes Dev..

[7] Delhommeau F (2009). Mutation in TET2 in myeloid cancers. N. Engl. J. Med..

[8] Ko M (2010). Impaired hydroxylation of 5-methylcytosine in myeloid cancers with mutant TET2. Nature.

[9] Yang H (2013). Tumor development is associated with decrease of TET gene expression and 5-methylcytosine hydroxylation. Oncogene.

[10] Kudo Y (2012). Loss of 5-hydroxymethylcytosine is accompanied with malignant cellular transformation. Cancer Sci..

[11] Lian CG (2012). Loss of 5-hydroxymethylcytosine is an epigenetic hallmark of melanoma. Cell.

[12] Turcan S (2012). IDH1 mutation is sufficient to establish the glioma hypermethylator phenotype. Nature.

[13] Bachman M (2014). 5-Hydroxymethylcytosine is a predominantly stable DNA modification. Nat. Chem..

[14] Pfeifer M (2013). PTEN loss defines a PI3K/AKT pathway-dependent germinal center subtype of diffuse large B-cell lymphoma. Proc. Natl Acad. Sci. USA.

[15] di Martino E, L’Hote CG, Kennedy W, Tomlinson DC, Knowles MA (2009). Mutant fibroblast growth factor receptor 3 induces intracellular signaling and cellular transformation in a cell type- and mutation-specific manner. Oncogene.

[16] Parish A (2015). Fibroblast growth factor family aberrations in cancers: clinical and molecular characteristics. Cell Cycle.

[17] Yin Y (2013). Rapid induction of lung adenocarcinoma by fibroblast growth factor 9 signaling through FGF receptor 3. Cancer Res..

[18] Zhu L (2005). Fibroblast growth factor receptor 3 inhibition by short hairpin RNAs leads to apoptosis in multiple myeloma. Mol. Cancer Ther..

[19] Herrera-Abreu MT (2013). Parallel RNA interference screens identify EGFR activation as an escape mechanism in FGFR3-mutant cancer. Cancer Discov..

[20] Hanahan D, Weinberg RA (2011). Hallmarks of cancer: the next generation. Cell.

[21] Jebar AH (2005). FGFR3 and Ras gene mutations are mutually exclusive genetic events in urothelial cell carcinoma. Oncogene.

[22] Jang JH (2000). Novel transcripts of fibroblast growth factor receptor 3 reveal aberrant splicing and activation of cryptic splice sequences in colorectal cancer. Cancer Res..

[23] Langemeijer SM (2009). Acquired mutations in TET2 are common in myelodysplastic syndromes. Nat. Genet..

[24] Kraus TF (2015). Genetic characterization of ten-eleven-translocation methylcytosine dioxygenase alterations in human glioma. J. Cancer.

[25] Yamazaki J (2015). TET2 mutations affect non-CpG island DNA methylation at enhancers and transcription factor-binding sites in chronic myelomonocytic leukemia. Cancer Res..

[26] Haffner MC (2011). Global 5-hydroxymethylcytosine content is significantly reduced in tissue stem/progenitor cell compartments and in human cancers. Oncotarget.

[27] Jin SG (2011). 5-Hydroxymethylcytosine is strongly depleted in human cancers but its levels do not correlate with IDH1 mutations. Cancer Res..

[28] Dang L (2009). Cancer-associated IDH1 mutations produce 2-hydroxyglutarate. Nature.

[29] Yan H (2009). IDH1 and IDH2 mutations in gliomas. N. Engl. J. Med..

[30] Ko M (2013). Modulation of TET2 expression and 5-methylcytosine oxidation by the CXXC domain protein IDAX. Nature.

[31] Wang Y, Zhang Y (2014). Regulation of TET protein stability by calpains. Cell Rep..

[32] Arechederra M (2018). Hypermethylation of gene body CpG islands predicts high dosage of functional oncogenes in liver cancer. Nat. Commun..

[33] Baylin SB, Herman JG (2000). DNA hypermethylation in tumorigenesis: epigenetics joins genetics. Trends Genet..

[34] Ushijima T, Nakajima T, Maekita T (2006). DNA methylation as a marker for the past and future. J. Gastroenterol..

[35] Solary E, Bernard OA, Tefferi A, Fuks F, Vainchenker W (2014). The Ten-Eleven Translocation-2 (TET2) gene in hematopoiesis and hematopoietic diseases. Leukemia.

[36] Schlessinger J (2000). Cell signaling by receptor tyrosine kinases. Cell.

[37] Marshall CJ (1995). Specificity of receptor tyrosine kinase signaling: transient versus sustained extracellular signal-regulated kinase activation. Cell.

[38] Boilly B, Vercoutter-Edouart AS, Hondermarck H, Nurcombe V, Le Bourhis X (2000). FGF signals for cell proliferation and migration through different pathways. Cytokine Growth Factor Rev..

[39] Jin Z (2017). Apatinib inhibits angiogenesis via suppressing Akt/GSK3beta/ANG signaling pathway in anaplastic thyroid cancer. Cell Physiol. Biochem..

