# Supplementary material for: FGFR3△7–9 promotes tumor progression via the phosphorylation and destabilization of ten-eleven translocation-2 in human hepatocellular carcinoma
Source: Cell Death Dis. 2020 Oct 23;11(10):903. doi: 10.1038/s41419-020-03089-2 (PMC7584635; doi:10.1038/s41419-020-03089-2)
Supplement: Supplementary file 1 — Supplementary Figure Legends [file 41419_2020_3089_MOESM1_ESM.doc]

**Supplementary Figure Legends**

**Supplementary Figure S1. PTEN is expressed at low levels in hepatocellular carcinoma tissues and is associated with poor prognosis.** **A**. Downregulation of PTEN in several cancer types in the TCGA database; **B.** R2 platform analysis was performed to detect the mRNA expression of PTEN and the overall survival of human HCC patients. **C and D.** The relations of protein level of PTEN and the overall survival or disease free survival in human HCC patients were analyzed through the TRGAted: https://nborcherding.shinyapps.io/TRGAted/).

**Supplementary Figure S2.** Western blot analysis for TET2, PTEN, AKT, and p-AKT in SMMC-7721/FGFR3∆7-9 and HepG2/FGFR3∆7-9 with indicated treatment.

**Supplementary Figure S3. A.** PTEN methylation level. **B.** The relation of PTEN methylation level and PTEN expression. **C.** Overall survival curves.

**Supplementary Figure S4. A.** The 5-hmC content at the PTEN promoter. **, P < 0.01. **B.** Overexpression of TET2 promotes demethylation of PTEN promoter region.

**Supplementary Figure S5.** CCK-8 cell proliferation assay for cells treated with DMSO and Wortmannin.

**Supplementary Figure S6. TET2 mutation is rare in HCC. A.** TET2 mutation status in several cancers; **B.** TET2 mutation status in HCC.
