# Supplementary figures and images for: FGFR3△7–9 promotes tumor progression via the phosphorylation and destabilization of ten-eleven translocation-2 in human hepatocellular carcinoma
Source: Cell Death Dis. 2020 Oct 23;11(10):903. doi: 10.1038/s41419-020-03089-2 (PMC7584635; doi:10.1038/s41419-020-03089-2)

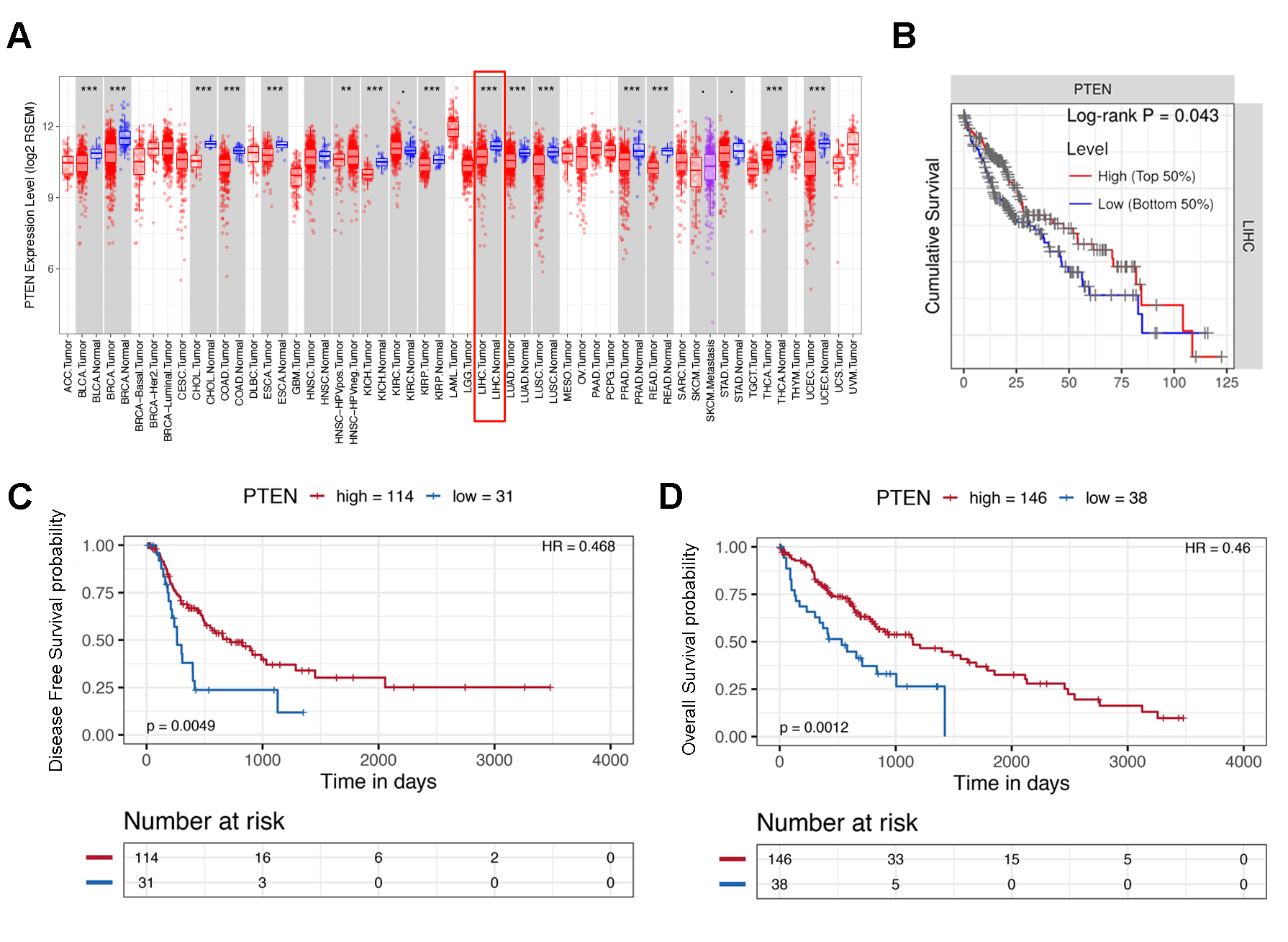

Supplement: Supplementary file 2 — Supplementary Figure 1 [file 41419_2020_3089_MOESM2_ESM.tif]

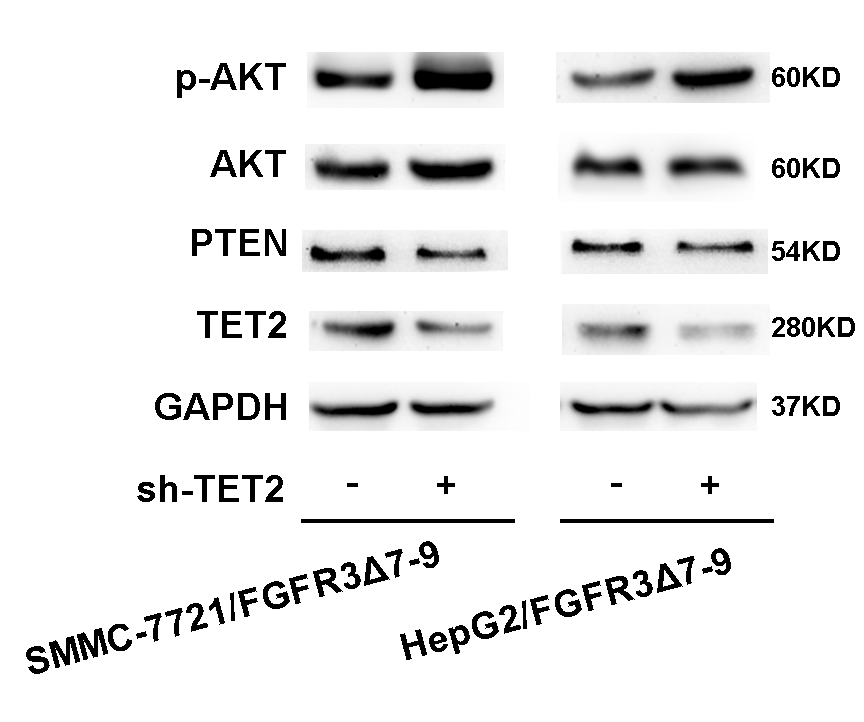

Supplement: Supplementary file 3 — Supplementary Figure 2 [file 41419_2020_3089_MOESM3_ESM.tif]

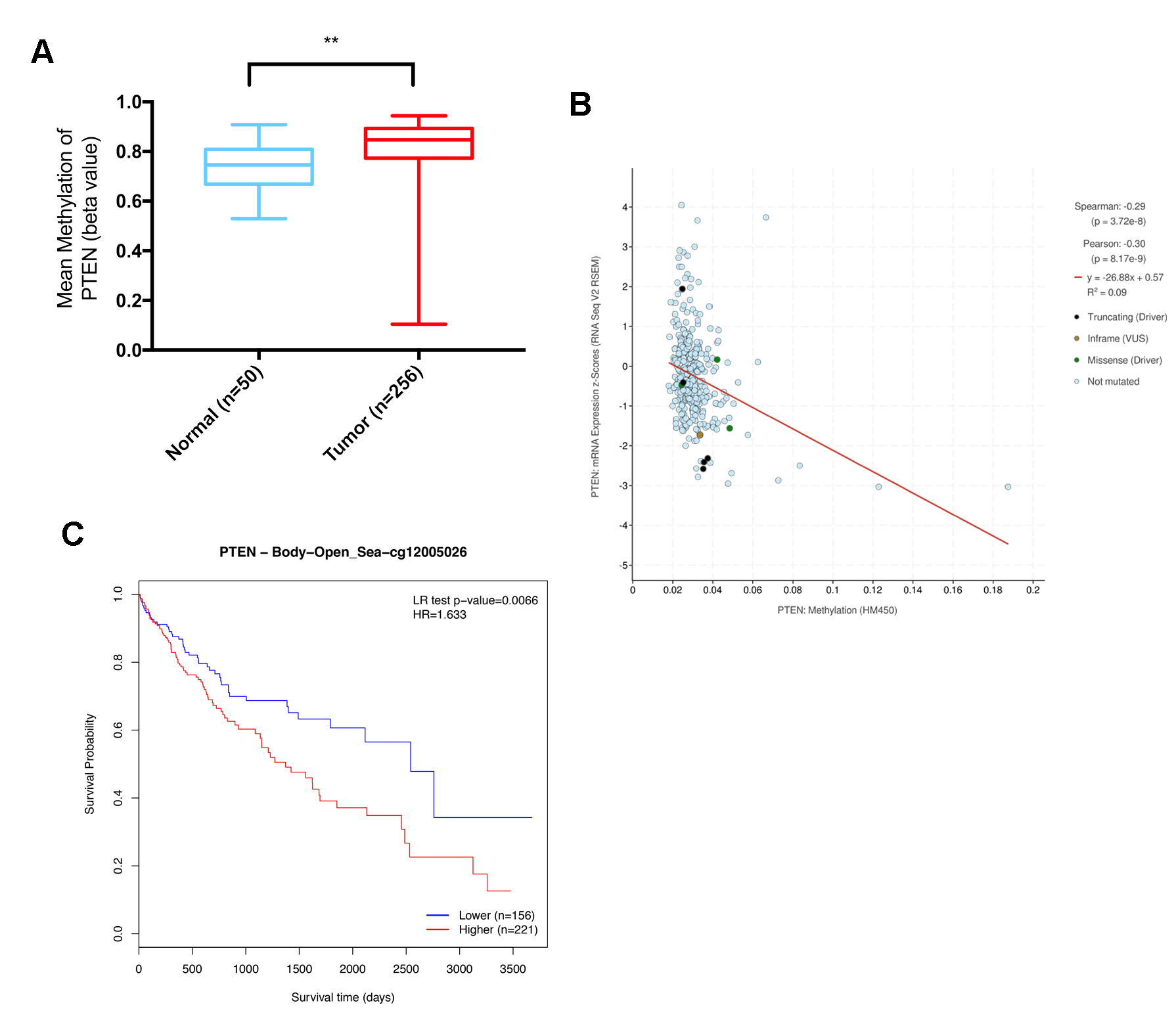

Supplement: Supplementary file 4 — Supplementary Figure 3 [file 41419_2020_3089_MOESM4_ESM.tif]

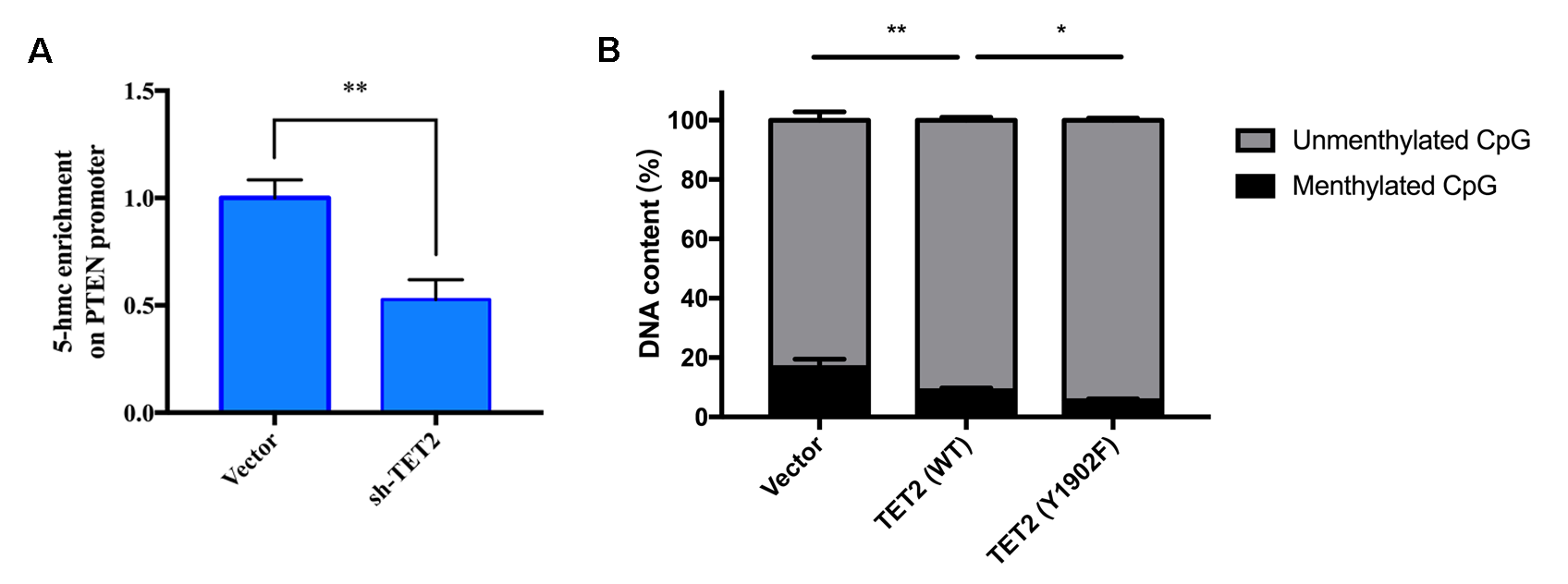

Supplement: Supplementary file 5 — Supplementary Figure 4 [file 41419_2020_3089_MOESM5_ESM.tif]

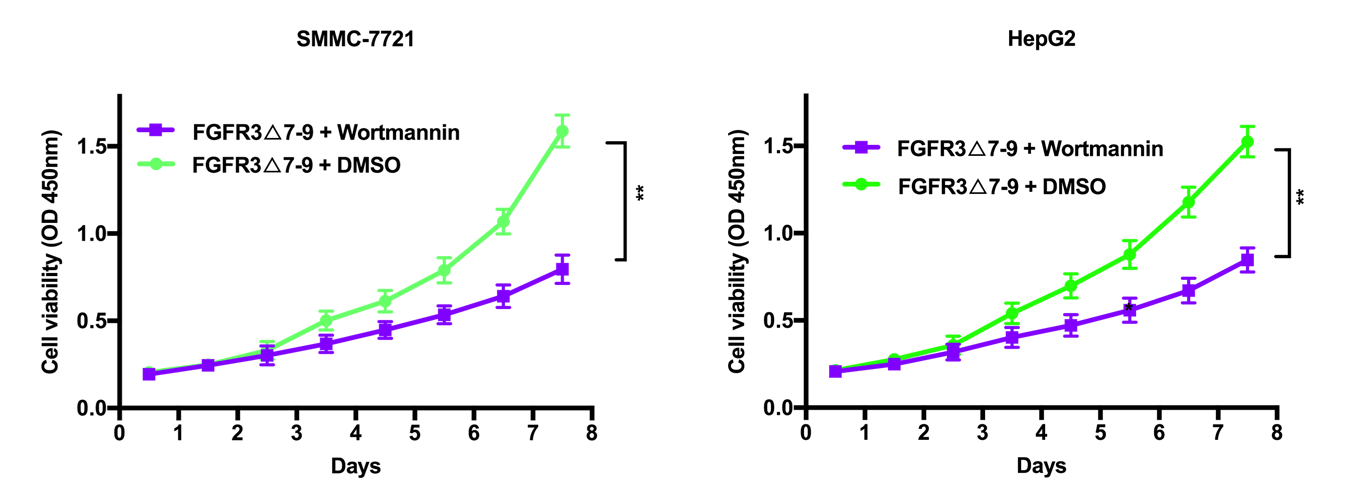

Supplement: Supplementary file 6 — Supplementary Figure 5 [file 41419_2020_3089_MOESM6_ESM.tif]

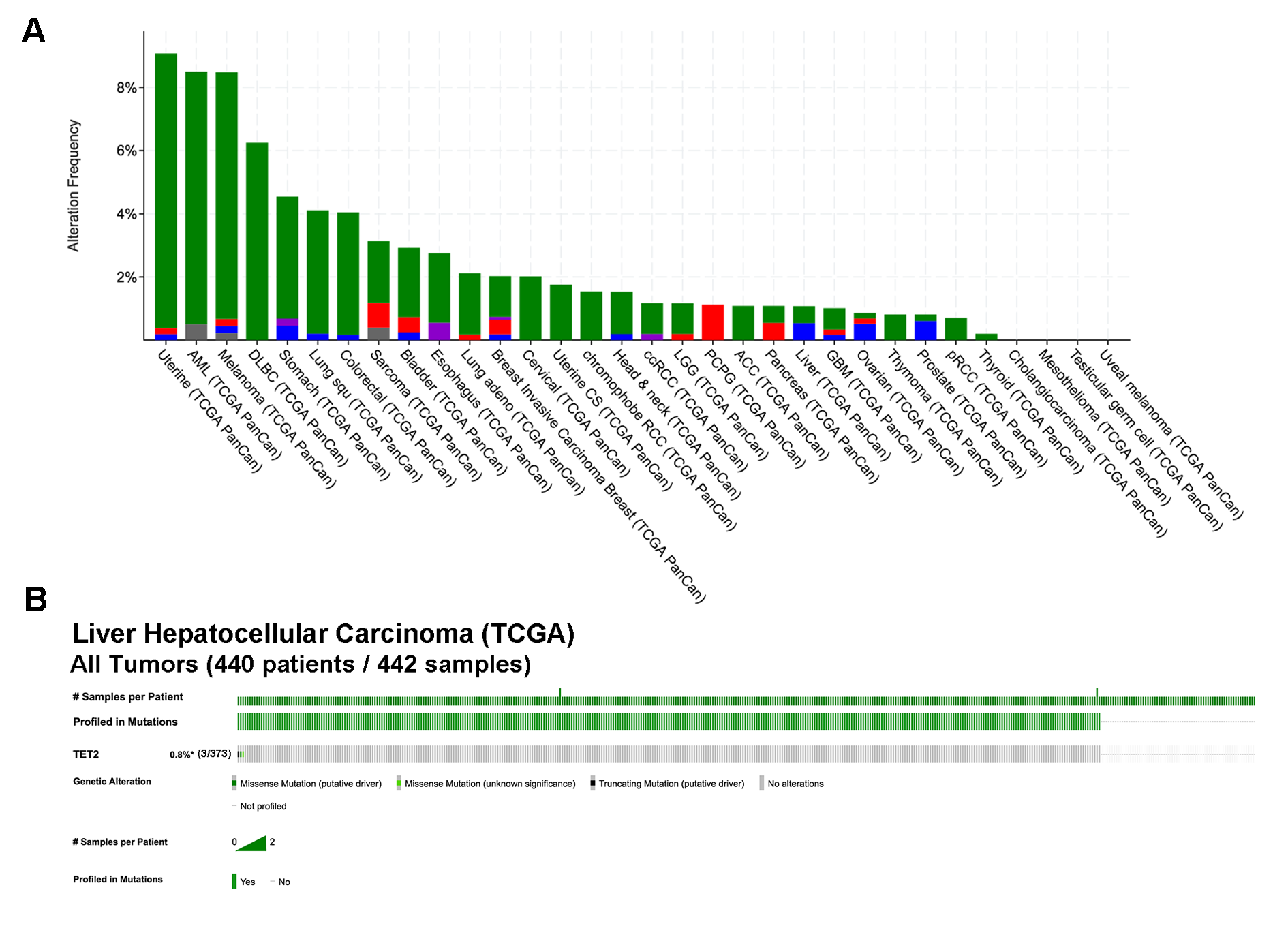

Supplement: Supplementary file 7 — Supplementary Figure 6 [file 41419_2020_3089_MOESM7_ESM.tif]
