# Supplementary material for: FGFR3△7–9 promotes tumor progression via the phosphorylation and destabilization of ten-eleven translocation-2 in human hepatocellular carcinoma
Source: Cell Death Dis. 2020 Oct 23;11(10):903. doi: 10.1038/s41419-020-03089-2 (PMC7584635; doi:10.1038/s41419-020-03089-2)
Supplement: Supplementary file 8 — Supplementary Table S1. [file 41419_2020_3089_MOESM8_ESM.docx]

**Supplementary Table S1.**

| Primer site | sequece (5'-3') |
| --- | --- |
| **TET2 primer for qPCR** |  |
| Forward | ATACCCTGTATGAAGGGAAGCC |
| Reverse | CTTACCCC GAAGTTACsGTCTTTC |
| **PTEN primer for qPCR** |  |
| Forward | CCCAAGCAACTAGCCCCTC |
| Reverse | GGCAGCACATCAGGGTAGTC |
| **GAPDH primer for qPCR** |  |
| Forward | AGCCACATCGCTCAGACAC |
| Reverse | GCCCAATACGACCAAATCC |
| **PTEN promoter set1 primer for ChIP-qPCR** |  |
| Forward | CTCACCAGCTCAGGGGTAGT |
| Reverse | CCCTTTTGGTTCTGTGCTTG |
| **PTEN promoter set2 primer for ChIP-qPCR** |  |
| Forward | CCCGTGTATCCTTCCACCTC |
| Reverse | TGGAAAGTACGGAACGGTAGG |
| **PTEN promoter set3 primer for ChIP-qPCR** |  |
| Forward | GTGCAAAAGGAAAGAGCGAA |
| Reverse | ACGCTGCTCAGTGTAGAGGG |
| **PTEN primer for GlucMS-qPCR analysis** |  |
| Forward | CTATGTGTTCACGTTCAGCACG |
| Reverse | CTAG AGATTCCCCCTTCCCC |
